# Death of Dr. Warren, with Sketch of His Character, &c.

**Published:** 1836-01-01

**Authors:** 


### Dr. Warren.

In addition to the death of Sir D. Barry, we grieve to announce the decease of Dr. Warren—at the age of 58, by organic disease of the liver. We have long been professionally acquainted with Dr. Warren, and we have always found him a man of upright and honourable character. He was not so mild and polished in his manners as Dr. Maton; nor was he so punctual in his engagements; but he never took the advantage of his name and rank in the profession, to hurt the feelings of his junior brethren, or deprive them of the confidence of patient or friends. We wish we could say as much for all the medical magnates!

Dr. W. was a shrewd practitioner, of great experience; but one who had left the improvements of the last twenty, or even thirty years, in almost total neglect. Modern pathology he either despised, or left comparatively unheeded. The *latter* we believe to be nearest to the truth. If he ever adverted to any recent discovery or improvement (we mean in anatomy, physiology, pathology, or diagnosis) it was to afford matter for a joke or a sarcasm, in his dry and caustic manner. He had a good deal of humour about him, and every ebullition of it was preceded by an unusually large pinch of snuff. The ordinary doses, however, of this weed were great and frequent—the daily consumption must have been prodigious! His dress was slovenly—and his linen seldom redounded to the reputation of his laundress. The plainness of his exterior corresponded very much with his moral manners. The meanest observer became convinced, in the course of his observations, that he was destitute of—HUMBUG. This very circumstance greatly enhanced his reputation. There is not a more erroneous notion in existence than a prevalent one, that the public may be humbugged *ad infinitum*, by dextrous skill on the part of the medical practitioner. The trickery lasts for a space of time corresponding with the talent of the actor; but the bubble bursts sooner or later, and demonstrates the truth, that nothing but honesty, ability, and industry will finally triumph in the practice of medicine.

Honesty, and a fair share of natural ability, sustained Dr. Warren in extensive practice till the end of his life—certainly without the aid of any other industry than that of attending his patients, and collecting his fees. He has left nothing on record that has conduced to the benefit of his profession, or that can float his name, even for a month, on the tide of time. This was more owing, perhaps, to the class than to the individual. There is a certain proportion (we do not say a majority) of those who have imbibed large potations of aristocratic pride and port on the banks of the Cam and the Isis, and who are afterwards so wrapt up in their own dignity, that they shrink from collision with their plebeian brethren, and abhor any other competition than that which is connected with the GUINEA TRADE. It is true that, among this section of the CANTABS and OXONIANS, a few are goaded, by the stings of penury, to enter the field of science, and manfully compete with high and low in the ranks. We regret to say that some, even of these, when the pressure of adversity is withdrawn, shrink back within the narrow sphere of their own importance, ashamed, as it were, of having exposed their ORDER to contamination with the vulgar herd, and to the shafts of criticism or ridicule during the contest.

These reflections are not levelled at our deceased contemporary. Dr. Warren was any thing but a proud man—any thing but a courtier. He was a prickly pear outside, but a pine-apple within. That INDOLENCE which prevented him from taking any but the passive exercise of a close carriage, and which, doubtless, led to the formation of those organic changes which terminated his life, was probably the cause why he carried with him to the grave a mine of valuable facts and observations, which would have enriched the archives of medical science, had they been communicated to the world.

It is surely the bounden duty of every man who has been carried forward to riches and reputation, in the medical profession, to repay a part of the debt which he owes to that profession and the public, by contributing something to the good of both. The excuse which we have heard made, that medical literature is oppressed by a host of sciolists and scribblers, is any thing but valid. This is the very best of reasons why men of talent, and wide experience should diffuse the results of their observations among their brethren, in order to correct, or at least, neutralize the deleterious effects of false facts and futile theories. He who does not adopt and act upon this precept and principle, may accumulate a princely fortune, but he dies a bankrupt in science, and a debtor to the community.

